# Donor cross-linking for keratoplasty: a laboratory evaluation

**DOI:** 10.1007/s00417-015-3160-6

**Published:** 2015-09-08

**Authors:** Achyut Mukherjee, Sally Hayes, Ioannis Aslanides, Elena Lanchares, Keith M. Meek

**Affiliations:** School of Optometry and Vision Sciences, Cardiff University, Wales, UK; Emmetropia Mediterranean Eye Institute, Heraklion, Greece; Essex County Hospital, Colchester, Essex, UK

**Keywords:** Keratoplasty, Corneal cross-linking, Riboflavin/UVA cross-linking

## Abstract

**Purpose:**

This laboratory-based investigation compares the topographic outcomes of conventional penetrating keratoplasty with that of a novel procedure in which donor corneas are cross-linked prior to keratoplasty.

**Methods:**

Penetrating keratoplasty procedures with continuous running sutures were carried out in a porcine whole globe model. Sixty eyes were randomly paired as ‘donor’ and ‘host’ tissue before being assigned to one of two groups. In the cross-linked group, donor corneas underwent riboflavin/UVA cross-linking prior to being trephined and sutured to untreated hosts. In the conventional keratoplasty group, both host and donor corneas remained untreated prior to keratoplasty. Topographic and corneal wavefront measurements were performed following surgery, and technical aspects of the procedure evaluated.

**Results:**

Mean keratometric astigmatism was significantly lower in the cross-linked donor group at 3.67D (SD 1.8 D), vs. 8.43 D (SD 2.4 D) in the conventional keratoplasty group (*p* < 0.005). Mean wavefront astigmatism was also significantly reduced in the cross-linked donor group 4.71 D (SD 2.1) vs. 8.29D (SD 3.6) in the conventional keratoplasty group (*p* < 0.005). Mean RMS higher order aberration was significantly lower in the cross-linked donor group at 1.79 um (SD 0.98), vs. 3.05 um (SD 1.9) in the conventional keratoplasty group (*P* = 0.02). Qualitative analysis revealed less tissue distortion at the graft-host junction in the cross-linked group.

**Conclusion:**

Cross-linking of donor corneas prior to keratoplasty reduces intraoperative induced astigmatism and aberrations in an animal model. Further studies are indicated to evaluate the implications of this potential modification of keratoplasty surgery.

## Introduction

Corneal transplant or keratoplasty procedures are well recognised in the management of axial opacity of the cornea to restore vision [1]. Both penetrating keratoplasty (PKP), where the full thickness of the cornea is replaced, and deep anterior lamellar keratoplasty (DALK), where only the anterior corneal layers are replaced, are established and successful procedures. However, the visual and refractive outcomes are suboptimal, with only about one third of PKP achieving 6/6 or better corrected vision [1–4]. In the case of DALK these outcomes in large series appear slightly worse or at best comparable [2, 5]. The dominant cause of limited visual outcome following PKP and DALK is the induction of regular and irregular astigmatism [3, 4, 6]; this has been elegantly demonstrated by the improvement in vision granted by adaptive optical correction [7]. Various refinements of surgical technique over the past decades have been developed to address keratoplasty astigmatism, with varying success. Subsequent surgical or laser correction of irregularity may also be performed [8]. A proportion of cases benefit from the use of rigid contact lenses to correct irregularities of the anterior refracting surface [8]. However, even with these interventions, visual limitation remains significant [7, 8].

Corneal cross-linking using UVA-activated riboflavin is a relatively recent but established procedure for the prevention of progression of corneal ectasia due to keratoconus [9]. The treatment, which stiffens the corneal stroma by induction of chemical cross-links, has been revealed to prevent progression in the majority, as well as improve topographic parameters in a subset of keratoconic corneas [10]. Within the limitations of these studies, corneal cross-linking appears to have a significant and lasting effect on corneal biomechanical parameters.

The induction of regular and irregular astigmatism in the visual axis of transplanted donor corneas is a function of a number of biomechanical considerations, particularly related to the alignment and tension along the point of attachment to the donor, i.e., the graft-host interface. Any mismatch between the shape of donor and host trephination will result in induction of irregularity [8, 11, 12]. Sutures are used to approximate the interface but may vary in tension, length, depth, alignment and spacing. Each of these variables at the graft-host junction in turn has an effect on the axial cornea by inducing irregular and regular astigmatism. In the case where the host cornea itself is irregular, particularly in ectasias, this further induces variable force and, hence, shapes at the visual axis of the donor [11, 12].

Given these considerations, we hypothesized that it might be expected that the induction of irregular and regular astigmatism within the donor cornea may be inversely related to the modulus of elasticity, in such a way that a more easily deformable donor cornea would be expected to exhibit higher levels of induced regular and irregular astigmatism than a stiffer donor cornea. If this were the case, we further hypothesized that increasing the stiffness of the donor cornea, by cross-linking it prior to keratoplasty, may result in a reduction of induced central regular and irregular astigmatism. If so, this novel procedure might provide a simple means to mitigate some of the visually limiting irregularity in corneal transplantation procedures.

## Materials and methods

The animal experiments in this paper comply with the Principles of Laboratory Animal Care (NIH publication No. 85–23, revised 1985), the OPRR Public Health Service Policy on the Humane Care and Use of Laboratory Animals (revised 1986), and the U.S. Animal Welfare Act, as amended, as well as specific national laws.

Sixty fresh porcine eyes were collected within 3 h of death from animals slaughtered for non-research purposes in an EC licensed abattoir (Maddock Kembrey Meats Ltd. Maesteg, UK). Thirty eyes were designated as ‘donor’ tissue and the remaining 30 eyes as ‘host’ tissue. The donor and host eyes were randomly paired and allocated into two groups. In the cross-linked group, the donor cornea was de-epithelialized and immersed in commercial riboflavin 0.1 % with dextran 20 % solution (Mediocross, Peshke Meditrade GmBH, Boesch, Switzerland), for between 30 min and 12 h. The donor eyes in the conventional keratoplasty group were also de-epithelialized and simultaneously immersed in 20 % dextran T500 solution (Pharmacosmos, Holbaek, Denmark). Host eyes were prepared identically to the conventional donor eyes.

All eyes were maintained in a light-excluded environment during this phase to prevent inadvertent cross-linking of the riboflavin-treated corneas. In keeping with the conventional protocol for in vivo cross-linking of keratoconus corneas [9], the cross-linked donor group was then exposed to 3 mW/cm^2^ UVA light for 30 min using a commercially available cross-linking lamp (CCL Vario Crosslinker, Peshke Meditrade GmBH, Boesch, Switzerland). During the cross-linking procedure, riboflavin eye drops were applied every 5 min. During the corresponding period, the donor corneas in the conventional keratoplasty group and the host corneas were maintained at ambient temperature and not exposed to any light.

**Fig. 1:**
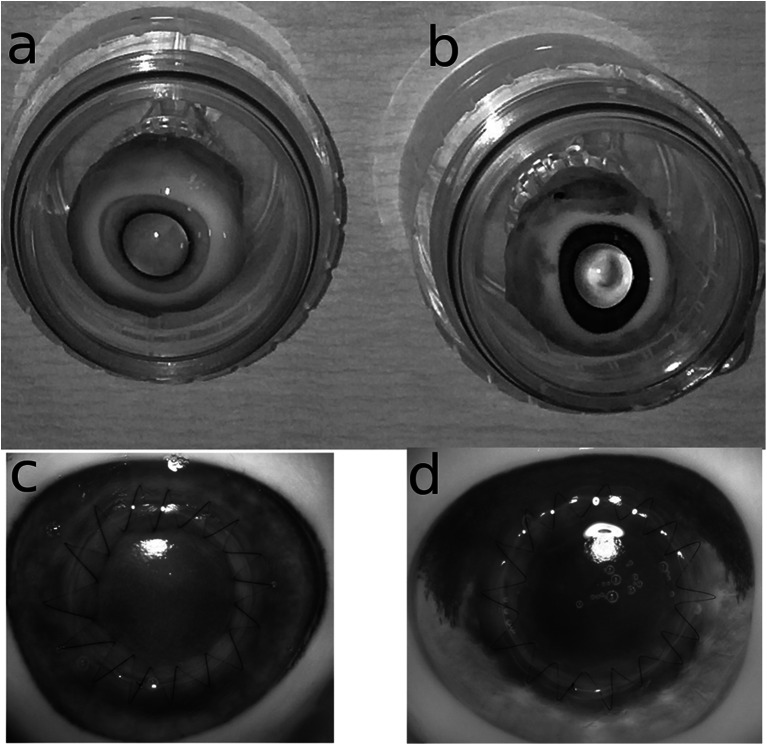
Photographs of a cross-linked (**a**) and conventional donor (**b**) globe prior to trephination, after cross-linked donor keratoplasty (**c**) and after conventional donor keratoplasty (**d**)

A keratoplasty procedure was subsequently performed for both groups (15 cross-linked donor keratoplasties and 15 standard untreated donor keratoplasties). All keratoplasty procedures in the study were carried out by a single surgeon (AM). Both donor and host globes were trephined using a 7.5 mm freehand corneal trephine and completed with corneal scissors. Sodium hyaluronate 1 % viscoelastic (Provisc, Alcon, Fort Worth, USA) was applied to maintain the anterior chamber and donors from both groups that were sutured using a standardised continuous single running 10/0 nylon suture with 16 bites under microscopic visualisation. No suture adjustment or keratoscopic visualisation was performed to avoid operator bias. In order to avoid distortion of topography induced by viscoelastic, this was aspirated via a small paracentesis and replaced with balanced salt solution (Alcon, Fort Worth, TX, USA). The globe was then inflated through this paracentesis to approximately physiological pressure determined digitally. The use of Tono-Pen tonometry (Reichert, NY, USA) was avoided due to concern that measurement itself might affect the corneal topography, while Goldmann applanation tonometry was not technically feasible. A small amount of artificial tear solution (hypromellose 0.3 %) was applied to the surface of each cornea to prevent drying and allow the reliable acquisition of topographic data. The typical appearance of eyes following cross-linked donor keratoplasty and conventional keratoplasty is shown in Fig. 1c and d, respectively.

Topography of the transplanted corneas was performed using a vertically mounted, intraoperative, high resolution, small cone placido topographer (Keratron Scout, Optikon SpA, Rome, Italy). To prevent distortion of the eyes during measurement, the globes were carefully mounted in a corneal viewing chamber (with the cornea facing upwards). The eyes were then rotationally aligned using the position of insertion of the extraocular muscles, and manually aligned with the red reflex to maintain axial orientation prior to measurement. Measurements were repeated six times for each specimen. The automated software was used to centre the horizontal alignment prior to capture. Following capture, the three readings with the least traceable ring images were discarded, and the remainder analysed. Automated analysis of topographic parameters and map were carried out, as well as corneal wavefront analysis of higher order aberrations including peak to valley (PV) and root mean square (RMS) values.

## Results

### Simulated keratometry

Simulated keratometry values derived from the topography were analysed. Mean Keratometric value was 30.9 D (SD 5.9 D; range 22 .8 to 44.5 D) in the cross-linked donor group and 34.6 D (SD 8.9 D; range 21.1 to 46.0 D) in the conventional keratoplasty group. There was no significant difference between groups (*P* = 0.209 *t*-test).

### Keratoplasty astigmatism

**Fig. 2:**
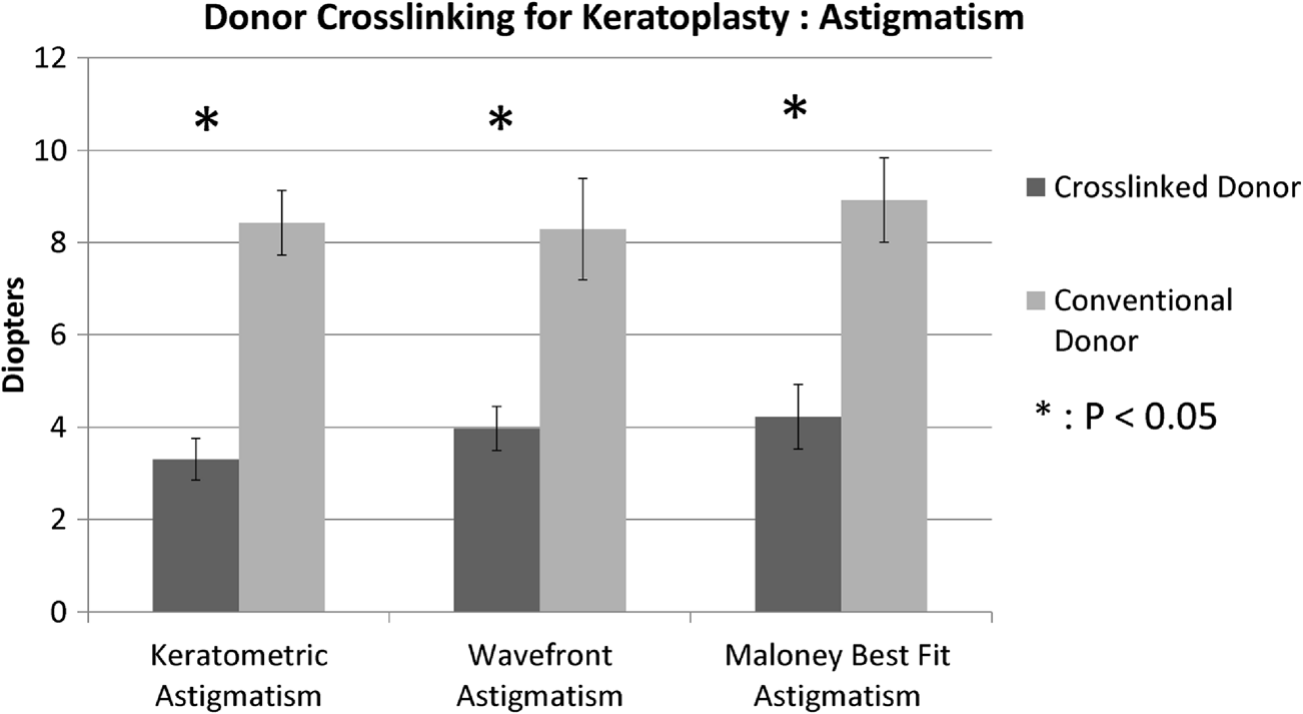
Graph comparing keratometric, wavefront, and Maloney best fit astigmatism between cross-linked donor corneas and conventional donors undergoing penetrating keratoplasty

Astigmatism values were derived from the simulated keratometry and from the wavefront measurement and compared between groups. Mean keratometric astigmatism was 3.67D (SD 1.8 D range 0.45 to 6.37 D) in the cross-linked donor group, and 8.43 D (SD 2.4 D, range 4.7 to 12.6 D) in the conventional keratoplasty group. Keratometric mean astigmatism was significantly lower in the cross-linked donor group (*p* < 0.005 *t*-test). Astigmatic measurement was also derived from the wavefront analysis, since this is a composite value derived across the assessment area, and may provide a more accurate assessment in highly irregular or aberrated corneas. Mean wavefront astigmatism was 4.71 D (SD 2.1; range 0.6 to 8.8) in the cross-linked donor group, and 8.29D (SD 3.6 D; range 2.1 to 14.7 D) in the conventional keratoplasty group. The mean wavefront astigmatism was also significantly lower in the cross-linked donor group (*p* < 0.005 *t*-test) (Fig. 2).

### Higher order aberrations

**Fig. 3:**
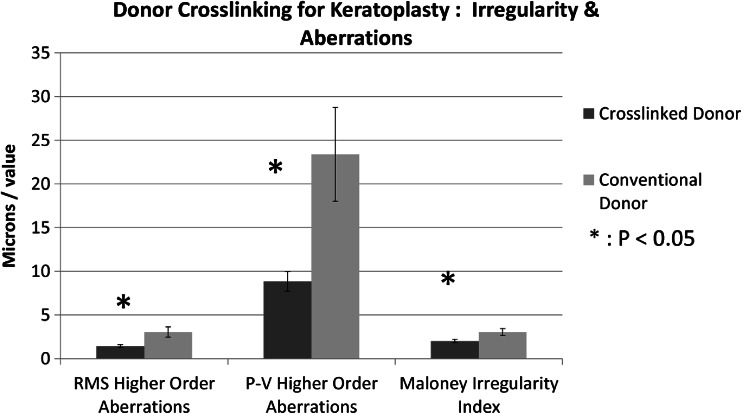
Graph comparing higher order aberrations (μm) and Maloney irregularity index between cross-linked donor corneas and conventional donors undergoing penetrating keratoplasty

The corneal wavefront measurement was performed using the inbuilt software of the topographer derived from the placido ring data. Centration of the corneal wavefront map was selected on the geometric centre of the donor as pupil-centered assessment was neither relevant to the current study nor technically feasible. Analysis was performed over an area of diameter 4.5 mm to assess the axial part of the donor cornea. Mean RMS wavefront aberration in the cross-linked donor group was 1.79 um (SD 0.98 range 0.67 to 4.34 um), while it was 3.05 um (SD 1.9 range 0.8 to 6.5 um) in the conventional keratoplasty group. RMS aberration was significantly lower in the cross-linked donor group (*P* = 0.02, *t*-test). Mean P-V aberration was 11.4 um (SD 6.7 range 4.1 to 29.9 um) in the cross-linked donor group and 23.4 um (SD 17.8 um range 5.3 to 57 um) in the conventional keratoplasty group, significantly lower in the cross-linked donor group (*P* = 0.029 *t*-test). Both coma and spherical aberration were independently analysed due to the visual relevance post keratoplasty. Mean coma aberration in the cross-linked and conventional keratoplasty groups was 1.01 um (SD 0.6, range 0.3 to 2.4 um) vs. 0.75 um (SD 0.67 range 0.1 to 2.3 um), while mean spherical aberration was −0.26 um (SD 0.5 range −1.4 to 0.3 um) vs. −0.09 um (SD 0.57 range −1.6 to 0.6 um), respectively. Neither coma nor spherical aberration differed significantly between groups (*P* = 0.3, *P* = 0.5, respectively) (Fig. 3).

### Qualitative analysis

**Fig. 4:**
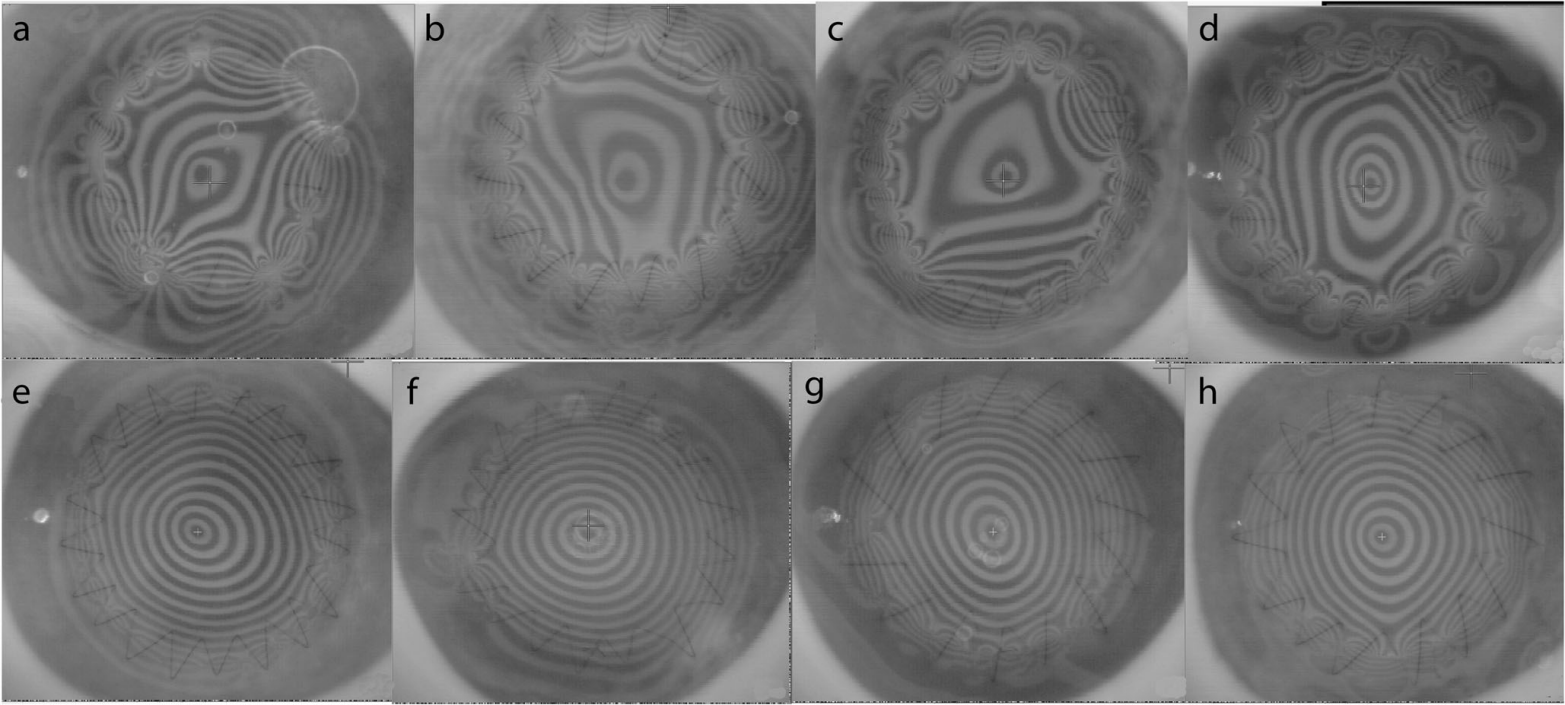
Representative placido ring images after penetrating keratoplasty with conventional donors (**a**–**d**) and cross-linked donors (**e**–**h**). A reduced area of distortion around the graft host interface is noted in cross-linked donors, with a more regular reflex and better quality ring images overall

Topographic maps were additionally analysed qualitatively. In particular, the pattern of ring images at the graft-host junction was evaluated since the reflex in this area was beyond the range of automated assessment by the topographer. There was a distinct dissimilarity noted between cross-linked and non-cross-linked donors. At the graft-host junction, the local distortion of placido reflex due to compression by each suture bite was a subjectively larger in terms of area and magnitude in the conventional keratoplasty donors, compared to the cross-linked donors. There was also a localised ring of steepening in a ‘doughnut’ shape, in the sutured area of conventional keratoplasty donors, which was largely absent in the cross-linked cases. A typical representative sample of ring images from conventional and cross-linked donors is presented in Fig. 4a–d and e–h, respectively.

### Surgical observations

Due to the altered appearance of the cross-linked donor corneas, the surgical operators could not be blinded to the donor group. A number of observations were made in relationship to the surgical aspects of keratoplasty in cross-linked versus conventional donors. Alignment and approximation of the sutured edges was much easier in the cross-linked group, since the tissue was more resistant to deformation and maintained shape during the passage of the needle. This was particularly noticeable during passage of the cardinal suture bites, where alignment can be challenging in keratoplasty procedures as the donor tissue is not yet fixed in place. There was a slightly increased resistance to the passage of the needle and suture in the cross-linked group, but this did not affect the ease of surgery. Achieving a watertight wound was also easier in the cross-linked group, since there was less distortion on application of suture tension and the wound was less affected by suture positioning and alignment. One negative aspect with the cross-linked donor keratoplasty procedure was the tendency to override the host tissue if vertically misaligned or over tightened, since, unlike in conventional keratoplasty donors, the compressed edge did not broaden under tension, while the host edge was deformed.

## Discussion

The primary objective of this study was to evaluate the possibility of reducing regular and irregular astigmatism and aberrations by cross-linking donor corneas prior to keratoplasty. To our knowledge this is the first report to evaluate corneal cross-linking of donor corneas for keratoplasty. The findings of this study support the hypothesis that altering the biomechanical rigidity of a keratoplasty donor may reduce the induced regular and irregular astigmatism of the axial refracting surface by lessening the effect of forces at the graft host junction.

We thus found significantly reduced values for all parameters evaluating regular astigmatism: simulated keratometry, as well as more composite geometric measures including Maloney best fit cylinder, and the reconstructed corneal wavefront astigmatism. All of these suggest that cross-linking the donor cornea may indeed reduce the regular astigmatism induced during a keratoplasty procedure. The magnitude of this effect was somewhat unexpected prior to commencing the study, since our expectation was that cross-linking would significantly reduce local changes in shape, by evenly distributing forces, and, thus, have an effect mainly on irregular astigmatism and high order aberrations, rather than reducing regular astigmatism. Nonetheless, the donor cross-linking procedure had a marked effect on regular astigmatism, with a reduction of less than half in all measured parameters. It is also noteworthy that no suture adjustment or intraoperative keratometry was performed, as would be usual in such procedures, thus, the level of astigmatism overall might be expected to be lower still if applied to clinical procedures.

Our primary motivation for developing the procedure however, was more to address irregular astigmatism and high order aberrations. These are a significant issue affecting visual outcomes of keratoplasty procedures, since they are not easily corrected and relate to the limited visual outcomes in a significant proportion of cases. RMS and P-V measures of HOA were significantly reduced to approximately one half and one third respectively of the values in the conventional keratoplasty group. The Maloney topographic irregularity index was also significantly lower in the cross-linked donors. These changes would be expected to lead to considerably better visual outcomes if mirrored in human keratoplasty procedures.

A secondary objective was to assess whether cross-linking would require any alteration of the surgical technique for keratoplasty. However the procedure required no significant changes to technique and was, if anything, easier to perform using cross-linked donors as alignment and placement of suture passes in more rigid tissue was simpler. The potential override of donor was easily avoided by attention to suture depth. In this regard, matched trephination techniques such as femtosecond laser trephination might be of particular benefit.

The main limitation of this study is the applicability of findings to a proposed human surgical procedure due to the biomechanical differences between human donor and porcine corneas [13]. Human donor corneas are often from older donors, and would be expected to be inherently more cross-linked physiologically. The porcine corneas, which came from animals between 6 months and 1 year old, appeared to have a lower rigidity than a typical human donor cornea in culture. It is, thus, unclear whether any additional biomechanical stiffening induced by cross-linking in human keratoplasty procedures would replicate the findings of this study. Human donor corneas are usually harvested as corneoscleral rims rather than whole globes, which may alter the corneal curvature, thus warranting planned further human donor cornea studies. The possibility that intraocular pressure variation affected the measurements to some degree cannot be excluded as accurate measurement was not performed for technical reasons. Nonetheless, there is no reason to expect any groupwise bias, nor would this factor be likely to explain the changes noted. A further potential bias is the un-blinded nature of the procedures, as it was not technically feasible to carry out keratoplasty without being aware of the cross-linked state. In order to limit the effects of this potential bias, the surgical protocol avoided any intraoperative keratometry or suture adjustment, limiting the effects of surgical bias on outcomes. The ultimate outcome of keratoplasty procedures in this regard can only be determined by an in vivo study following suture removal.

Although the effects of donor cross-linking on keratoplasty have not been evaluated previously, cross-linking has previously been applied in the setting of existing keratoplasty without concern. Spadea and Paroli investigated combined photorefractive keratectomy and cross-linking after lamellar keratoplasty in 14 eyes, with no noted adverse effect at a mean of 15 months [6]. Labiris et al. applied cross-linking to an infected penetrating keratoplasty with no noted adverse effect [14]. Kanellopoulous performed crosslinking of donor vehicle for Boston keratoprosthesis with no issues [15]. There may be potential concern for injury to the donor endothelium when cross-linking is applied to donor corneas. However, endothelial injury would theoretically be very unlikely as donor corneas are typically thicker than in the physiological state, and the procedure has been shown to be safe above 400 μm. Nonetheless, safety studies in human donor corneas would be advisable before any application of the study procedure to human keratoplasty. Keratoplasty has also been successfully carried out on previously cross-linked eyes [16]. A modified cross-linking procedure to enhance graft-host adhesion has previously been described in a laboratory model for femtosecond keratoplasty; however, this is unrelated to the technique we propose, since the donor was not cross-linked prior to keratoplasty and astigmatic outcome would be expected to be unaffected as the cross-linking is applied after graft alignment [17]. Cross-linking has also been utilized for biosynthetic corneal tissue engineering [18, 19]. In addition to the biomechanical effect, cross linking depletes keratocytes, and, thus, may theoretically have beneficial effects with regard to immunological rejection[20]. Clearly, further studies are warranted to ascertain whether the potential visual benefits of donor cross-linking for keratoplasty, as demonstrated here in the porcine eye model, may be replicated in human keratoplasty surgery.
